# Identification of pork in meat products using real-time loop-mediated isothermal amplification

**DOI:** 10.1080/13102818.2014.963789

**Published:** 2014-10-22

**Authors:** Lixia Yang, Shujun Fu, Xinkai Peng, Le Li, Taoping Song, Lin Li

**Affiliations:** ^a^ Changsha Center of Supervision & Inspection on Food Quality Safety, Yinshuang Road, Yuelu District, Hunan, Changsha410013, People's Republic of China

**Keywords:** real-time loop-mediated isothermal amplification, meat species identification, pork, mtDNA, cytochrome b

## Abstract

In this study, a one-step, real-time, loop-mediated isothermal amplification (RealAmp) assay was developed, for the highly specific detection of pork DNA. For the assay, the mtDNA of *cytochrome b* (*cytb*) gene was amplified at 63 °C using SYBR Green I for 45 min with a Real-Time Polymerase Chain Reaction (PCR) System that measured the fluorescent signal at one-minute intervals. As little as 1 pg of template DNA could be detected, without any cross-reactivity with non-target species. Meat mixtures, heat-treated at 100 °C for 15 min, prepared by mixing pork meat with beef at different ratios (0.01%–10%) were tested, and the RealAmp assays allowed the detection of as little as 0.01% pork in the meat mixtures. Thus, this work showed that RealAmp could be used for specific identification and sensitive quantification of meat species, even for heat-treated meat products.

## Introduction

The meat industry has become increasingly concerned about the substitution of low-priced meats such as pork, for higher priced meats such as beef and mutton, and the fraudulent labelling of meat species.[1] The identification of the animal origin of meat affects the food and meat industry, trade, markets, the restaurant industry and other fields.

The loop-mediated isothermal amplification (LAMP) method was developed as a novel method to amplify DNA specifically and simply.[2] LAMP employs autocycling strand displacement DNA synthesis using *Bacillus stearothermophilus* (Bst) DNA polymerase and a set of four primers that bind to unique sites on the target sequence, making them highly specific.[2] DNA amplifies efficiently under isothermal conditions (63–65 °C) with a detection limit of a few copies, eliminating the need for sophisticated, expensive thermal cyclers. Alternatively, the LAMP reaction can also be visually assayed by adding SYBR Green I dye to the reaction mixture; the colour of the solution changes to green in the presence of LAMP amplicons, but it remains orange in mixtures with no amplification.[3] LAMP has been used to detect bacteria,[4–6] viruses,[7–11] parasites,[12–14] and genetically modified organisms.[15–18]

The difficulty of visualizing precipitate, especially at lower target DNA concentrations, and high risk of cross-contamination, may limit the usefulness of the LAMP assay. The RealAmp method combines the amplification platform with a fluorescence detection unit, to acquire real time data,[19] and can produce a quicker and more objective readout for the LAMP method. RealAmp has been used for the diagnosis of malaria [19] and *Plasmodium vivax* infections.[20] In this study, we successfully developed and tested pork-specific LAMP primers using RealAmp on the 7500 fast real-time polymerase chain reaction (PCR) device.

## Materials and methods

### Preparation of meat mixtures

Tissues from the skeletal muscle of pork, cattle, sheep, chicken and duck were used. Meat mixtures, containing pork at 0.01%–10% (corresponding to 1 pg–1 ng target DNA), in a beef mixture were prepared and packaged in plastic bags. Patties of approximately 10 g were prepared for each mixture and subjected to heat treatment at 100 °C for 15 min in a water bath. Afterwards, DNA isolation was carried out on raw and heat-treated samples.

### DNA extraction

DNA was obtained from 500 mg of ground and homogenized sample according to Jain et al. [21] and the DNA concentration and purity were checked using a Biodropsis BD-1000 ultramicro nucleic acid protein analyser (Beijing Oriental Science & Technology Development Ltd., Beijing, China).

### Conventional PCR

To compare the sensitivity of conventional PCR to the established RealAmp assays, a conventional PCR was also performed. In our study, the size of the target sequence was 347 bp using the outer primers (F3 and B3) of the RealAmp system. The conventional PCR mixture (25 μL volume) included 2 × Mix (Tiangen Biotech (Beijing) Co., Ltd., Beijing, China), 0.5 μmol/L of each primer and template. The conventional PCR was performed in an S1000 thermal cycler (Bio-Rad Laboratories, Inc., USA) according to the following programme: initial denaturation at 95 °C for 5 min, 35 cycles of 94 °C for 30 s, 56 °C for 30 s and 72 °C for 30 s, and extension at 72 °C for 7 min. The products were analysed by 2% agarose gel electrophoresis in 0.5 × TBE with Goldview staining.

### Primer design for RealAmp

**Figure 1.**
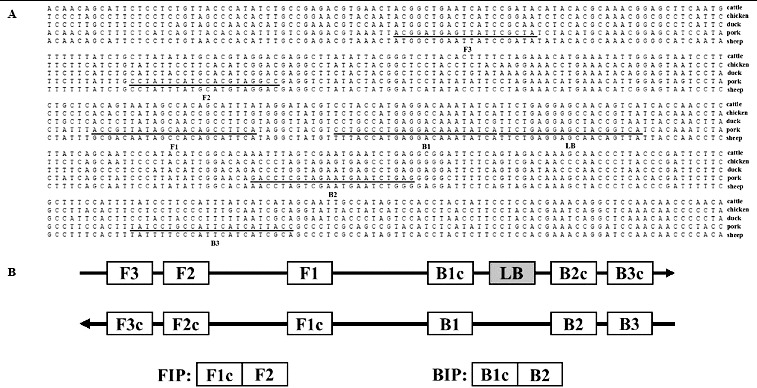
Primer design for RealAmp. (A) Nucleotide sequence alignment of the target regions of *cytb* genes. Arrows indicate the primers used for RealAmp assays. (B) Schematic diagram showing the positions of the RealAmp primers.

The pork RealAmp primers were designed using the mitochondrial *cytb* gene DNA sequence (GenBank accession no. X56295). The two outer (F3 and B3) and two inner (FIP and BIP) primers were designed using the online tool Primer Explorer Version 4.0 (http://primerexplorer.jp/elamp4.0.0/index.html) and the loop primer (LB) was designed manually (Figure 1). All the primers in this study were synthesized by Sangon (Shanghai, China) (Table 1).

**Table 1.** Nucleotide sequences of the primers (5′ → 3′).

Primer	Sequence
F3	ACGGATGAGTTATTCGCTA
B3	GGTAATGATGAATGGCAGGATA
FIP	TGAAGGCTGTTGCTATAACGGT-TTTT-CCTATTCATCCACGTAGGC
BIP	CCTGCCCTGAGGACAAATATCA-TTTT-CTCAGATTCATTCTACGAGGTC
LB	TTCTGAGGAGCTACGGTCA

### RealAmp method

The RealAmp method was performed using the commercially available DNA thermostatic amplification kit (Guangzhou Diao Bio-technology Co., Ltd., Guangdong, China) following the manufacturer's instructions. Reactions were performed in a 25 μL total volume containing 2 × reaction buffer (40 mmol/L Tris-HCl pH 8.8, 20 mmol/L KCl, 16 mmol/L MgSO_4_, 20 mmol/L (NH_4_)_2_SO_4_, 0.2% Tween-20, 0.8 mol/L Betaine, 2.8 mmol/L of dNTPs each), 0.5 μL of a 1:100 dilution SYBR green I (Invitrogen), 0.2 μmol/L of each outer primer (F3 and B3), 1.6 μmol/L of each inner primer (FIP and BIP), 0.8 μmol/L of loop primer of LB, 8 units of *Bst* DNA polymerase (New England Biolabs, Ipswich, MA) and 1 μL of template DNA. Amplifications were carried out at 63 °C for 45 minutes using the 7500 fast Real-Time PCR System (Applied Biosystems, USA) collecting fluorescence signals at one-minute intervals. All samples were run at least twice. If the two runs were discordant, the sample was run a third time and the two concordant results out of three runs were used to make the final call. We used a cut-off of 45 minutes; any sample that amplified after this cut-off point was considered to be negative. The specifically amplified products were first observed by eye, after adding 1 μL 1:10 dilution SYBR Green I into the reaction mixture. The reaction mixture will turn green in the presence of LAMP-amplified products, but it remains orange in a reaction mixture with no amplification. The products were detected by 2% agarose gel electrophoresis in 0.5 × TBE with Goldview staining after the RealAmp amplification.

## Results and discussion

### Optimization of the RealAmp assays

The aim of this work was to develop the RealAmp-based species-specific assays for the detection of pork DNA in meat samples. Species-specific PCR assays generally target the high-copy mtDNA,[22–27] which evolves much faster than nuclear DNA, thus providing sufficient sequence variation for the design of species-specific PCR primers.[28] In addition, mtDNA resists fragmentation by heat better than nuclear DNA. The *cytb*,[29–31] *COII* and [22] *ATPase 8* [32] genes have been used in pork species detection with mtDNA. We designed RealAmp primers using the pork mitochondrial *cytb* gene DNA sequence. The Blast alignment had been done for six Chinese native pig breeds (Bamei pig, Meishan pig, Ninggao pig, Qingping pig and Tongcheng pig), three Swedish domestic pig breeds (Landrace, Large white, Pietrain), Spanish pig breeds (Iberian), European wild boar and Asian wild boar. The results showed that the LAMP primer could be used for most of the pig breeds except for Landrace and Iberian pig breeds, which have one nucleotide substitution at nucleotide 14,529 and 14,596 of mtDNA, respectively.

**Figure 2.**
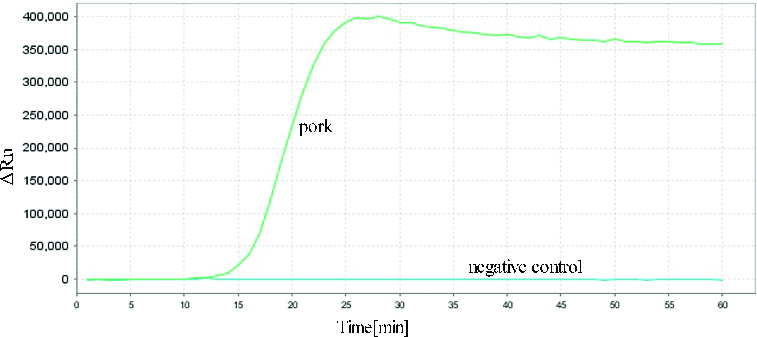
Real-time amplification of the pork *cytb* gene using the RealAmp assay.

**Figure 3.**
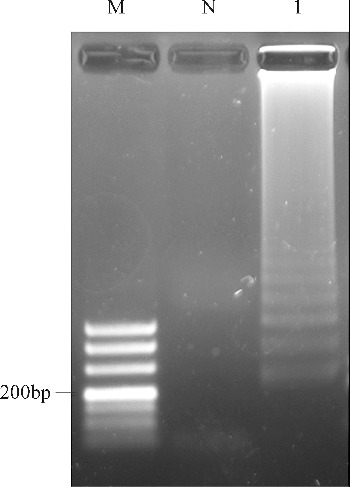
Analysis of pork *cytb* gene by RealAmp with agarose gel electrophoresis. Lane M, DL500 DNA Marker; lane N, negative control; lane 1, RealAmp products of *cytb*.

The reactions for pork *cytb* RealAmp were performed under isothermal conditions at 63 °C using 10 ng genomic DNA, for 60 min. No amplification was detected for the negative sample until after at least 45 min of incubation (Figure 2). Thus, subsequent RealAmp reactions were conducted at 63 °C for 45 min. A successful LAMP reaction was also detected by electrophoresis on a 2% agarose gel, as shown in Figure 3. The RealAmp products give a ladder-like pattern on the agarose gel, due to their characteristic structure (Figure 3).

**Figure 4.**
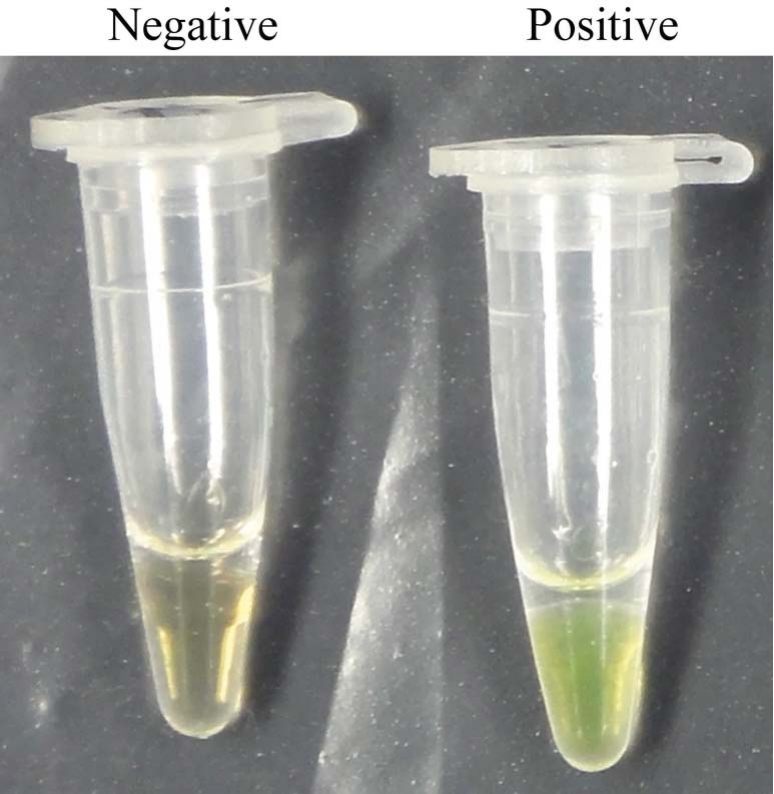
SYBR green I fluorescent dye-mediated monitoring of pork specific RealAmp assay amplification.

We also monitored RealAmp assay amplification by visual inspection following the addition of 1 μL of SYBR Green I dye to the amplified products (Figure 4). The positive tube displayed a visible green colour, whereas the negative tube maintained the orange colour of unbound SYBR Green I.

### Specificity of the RealAmp assay

**Figure 5.**
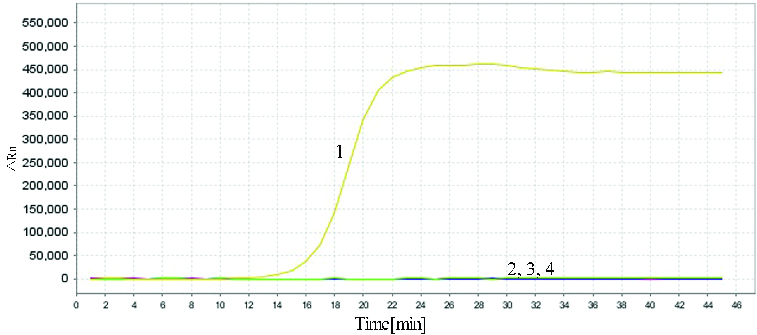
Specificity of RealAmp assays. Sample 1, pork; sample 2, cattle; sample 3, sheep; sample 4, chicken; sample 5, duck; sample 6, negative control.

To evaluate the specificity of the developed RealAmp assays, we used meat from four other species (cattle, sheep, chicken and duck). In the specificity test, 10 ng total genomic DNA was used as the template in each RealAmp assay. As expected, the typical amplification curve was only obtained in the test using pork DNA samples as template; no amplification was observed for other species or the negative control (Figure 5). The result showed that the RealAmp assays have high specificity, suggesting that it was suitable for the identification of the pork DNA.

### Sensitivity and linearity of RealAmp

**Figure 6.**
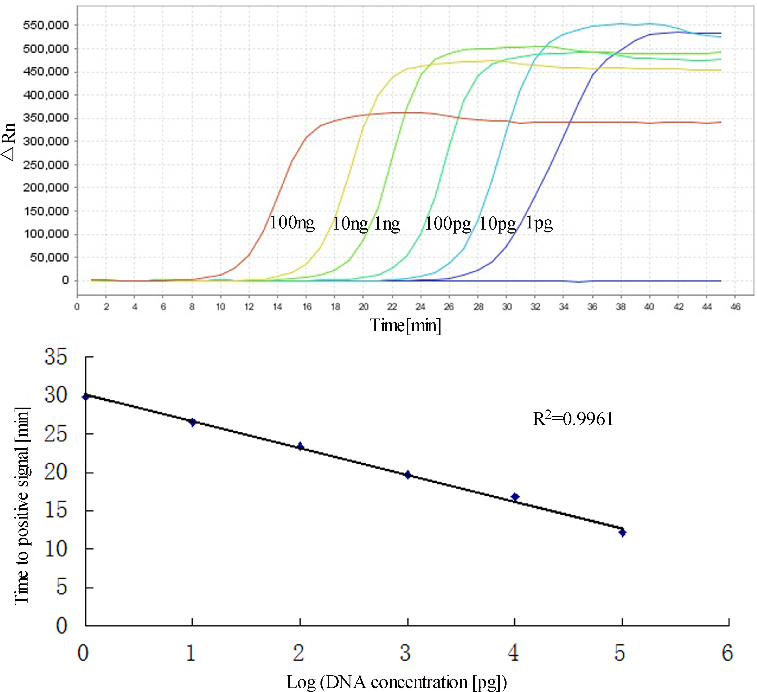
Sensitivities of RealAmp assays performed with serial dilutions of pork DNA (100 ng to 1 pg). (A) Amplification curves of RealAmp detection for pork DNA. (B) Standard curves for RealAmp assays generated from the amplification plots between serial 10-fold dilutions of pork DNA and the time to a positive signal by employing the specific RealAmp assay.

**Figure 7.**
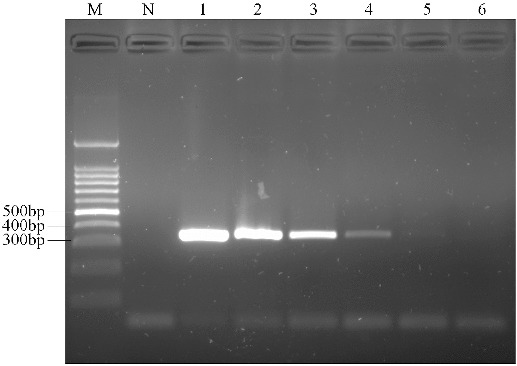
Agarose gel electrophoresis of PCR products targeting the pork *cytb* gene in different amounts of DNA. Lane M, 100 bp DNA ladder; lane N, negative control; and lanes 1–6, 100 ng, 10 ng, 1 ng, 100 pg, 10 pg, 1 pg pork DNA, respectively.

To ascertain the detection limit of RealAmp, we tested serial 10-fold dilutions of pork DNA starting with 100 ng, and compared the results with those of conventional PCR with the outer primers (F3 and B3). The RealAmp assay demonstrated higher sensitivity than the conventional PCR. The limit of detection of RealAmp was 1 pg/μL (Figure 6(A)), which was 100-fold more sensitive than PCR (100 pg/μL) (Figure 7). By real-time monitoring of the amplification of different concentrations of pork DNA ranging from 1 pg to 100 ng/μL, we constructed a standard curve depicting the linear relationship between the concentrations of DNA to the time that we could detect a positive signal (Figure 6(B)). We also added SYBR Green I after the reaction and the colours showed weak positive at 1 pg (data not shown).

### RealAmp analysis of pork in meat mixtures

We used the RealAmp method to analyse heat-treated beef mixtures containing pork meat at 0.01%, 0.1%, 1%, 10%, 100% and found that the detection limit was 0.01% (corresponding to 1 pg) (Table 2). The time to a positive signal, corresponding to the DNA concentration of the target species, showed high linearity over a wide range of template concentrations, with a correlation coefficient of 0.9965, which enables consistent and precise determination of target DNA in all tested meat mixtures. The presence of undeclared species below 0.1% in meat products is generally considered to result from accidental contamination, because low adulteration produces no economic benefit.[33] Therefore, the detection limit obtained using RealAmp is sufficient to quantitatively detect accidental contamination.

**Table 2.** Results of RealAmp measurement of heat-treated meat mixtures.

Assay target species	The ratio of target species in the binary mixtures (%)	Time to positive signal
Pork	100	21.28
	10	24.79
	1	28.58
	0.1	33.51
	0.01	36.91

The RealAmp qualitatively detected pork *cytb*, but also quantitatively showed the percentage of meat DNA in an unknown sample by interpolation from a standard curve of time of detection of positive signal, from known starting DNA concentrations.

The ready-to-use reaction buffer was employed for RealAmp amplification. This saved time, simplified standardization, and also increased the specificity, sensitivity, accuracy and repeatability. If it is combined with simple and fast DNA extraction methods, which would be further useful for meat detection in the laboratory and also on site.

## Conclusions

In this study, we developed a RealAmp technique for the specific and quantitative detection of pork in meat mixtures. Species-specific RealAmp primers were designed to target using the mitochondrial *cytb* gene. The detection limit in heat-treated meat samples using the RealAmp technique was 0.01%, which was sufficient to detect accidental contamination (<0.1%) in meat products. The RealAmp method provided quantitative data for detection purposes by a sensitive, practical and rapid method.
